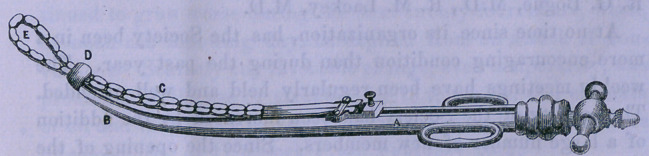# Removal of Uterine Polypus by a Modified Form of the Ecraseur

**Published:** 1867-06

**Authors:** Jonathan W. Brooks

**Affiliations:** Chicago, Ill.


### REMOVAL OF UTERINE POLYPUS BY A MODIFIED FORM OF THE ECRASEUR.

By Jonathan W. Brooks, M.D., Chicago, Ill.

The writer was first consulted by Miss E. L., of S—n, Wis., May 9th, 1866, for protracted uterine hemorrhage of an obscure character, which had existed for several months. Her age was about 35 years, and her form slender; but up to the November previous, had enjoyed uniform good health since her sixth year. A teacher by profession. At this time, the following conditions were present:—General anæmia; pulse small, irritable, and 100; she reports a constant flowing for the last five months; bowels torpid, and urine not free; faints often; walking is accompanied by faintings and palpitations; frequent nausea and general dyspeptic symptoms. Has been treated for menorrhagia. Objected to a tactile examination.

On *June 8th*, was consulted again by her, for alarming hemorrhage, which had occurred twelve hours previously. The general indisposition had perceptibly increased. A thorough examination was now permitted. The os uteri was very firmly closed, yet there was a stillicidium of grumous fluid, tenderness of the right half of the cervix uteri, and the body of the uterus seemed slightly enlarged. The diagnosis was a small polypus within the cavity of the uterus. Suggested absolute rest in the recumbent posture. ℞. Fluid ext. senna, fluid ext. taraxacum, āā ʒj. Take ʒj. at night as a laxative, *pro re nata*. ℞. Gallic acid ʒj., chart. No. xij. Take one every three hours, unless the hemorrhage ceases or the acid disagrees with the stomach; and dilation of the os with a tent. We were foiled in the last by her objection.

*July 10th.* By request, saw her again, with the assurance that whatever was proper might be done. Found the appetite somewhat improved, the hemorrhage not so great as the month which was completed seven days since, and less constipation. She complained of nearly constant pain in the hips, sacrum, and groins, with a sensation of weight in the uterine region. Tactile examination disclosed the os uteri as less rigid, but still was unable to gain access within the os for exploration with the finger. She now placed herself under my immediate care. A silver tent was, at this time introduced, which was removed at the expiration of four hours. The next day it was repeated, and on the third day I had the satisfaction of confirming the original diagnosis, by discovering a small polypus attached to the interior, superior, and posterior part of the fundus of the uterus, not far from the orifice of the right Fallopian tube. The catamenia appeared the same evening, at which time the polypus could be touched without difficulty. Hemorrhage being added to the secretion of the monthly molimen, directed the following:—℞. Opii pulv., camphor pulv., ipecac. pulv., āā grs. iij., sacch. saturni grs. xij. M. Chart. No. viij. Take one every two hours till the hemorrhage abates, which it did in twelve hours, and the catamenial secretion ceased on the sixth day. An examination announced that the os had firmly closed again, and, the hemorrhage being stayed, I determined to delay farther proceedings, to await developments, and examine two or three times during the month, believing from past observation that a few days preceding the secretion of the monthly molimen would enable better to establish the former observations. On the *27th of July* the relaxation of the os commenced, on the *28th* the polypus could be touched, and on the *30th* the secretion appeared. She had improved considerably, the hemorrhage was less than at the last period, and on the *3d of August* both ceased. On the *7th of August* the os was fully and firmly closed. She desired to wait a time before operative measures were taken, first, because she was suffering much from the heat of the weather, and, second, her health would, perhaps, improve more, to which I assented.

On the *20th of August*, indications of the monthly secretion appeared. On examination, found the os dilated to the size of a large quill; easily dilated farther, and the polypus examined with less difficulty. This period passed over much as the last, and the os was closed fully on the third day after the cessation, which occurred *August 28th.*

The month of September was passed very comfortably by her, she still being kept in the recumbent posture. On the *15th of September* the os was dilatable, and the polypus could be reached with less difficulty. On the evening of the *23d*, the catamenia appeared. There was less hemorrhage, and her health was slightly improved.

The case, from this time, was carefully observed, and on the thirteenth day from the commencement of the secretion of the monthly molimen and the seventh from its close, which was *Oct. 6th,* I determined to try to remove the offending cause, assisted by the skillful and excellent J. D. Skeer, M.D., late surgeon in the U.S. Army. The patient being placed on the left side, the chest rotated forward, the arm thrown across the back, the thighs flexed at right angles with the pelvis, the right one drawn up a little more than the left, and a small pillow between the knees. Being supplied with a univalve speculum, an ecraseur, modified from Chassaingnac, and small forceps, somewhat resembling Dr. McClintock's, of Dublin, we proceeded to dilate the vulva and vagina with the speculum, the os with the forceps, and, with the aid of the ecraseur, soon removed a small fibro-cellular polypus, which was covered with a thin, glossy membrane which gave way during the operation.

From this membrane, in my opinion, proceeded the hemorrhage. No hemorrhage occurred at the time of removal, nor has any since that time. The size and shape of the polypus was not unlike a medium sized hickory nut. She was placed in bed and kept quiet a few days; no untoward symptoms followed. On the *18th of October*, being the eleventh full day from the operation, she returned to her home in S——n, Wis. Recently, she informed me that she was quite well, and that the catamenia have returned regularly and naturally, and that she expects to resume teaching the first week in January, 1867.

In several cases of small polypi interna, I have found the polypus apparently to descend just prior to the secretion of the monthly molimen, and recede with the cessation of it, the os closing somewhat firmly at the same time and remaining so until the approach of the next catamenial period, rendering the diagnosis difficult, especially to a novice. Observation has convinced me that, ordinarily, the best time for removing them, when the menstrual secretion continues, is early after the secretion has ceased, for the reason that the engorgement of the uterine organs, and the irritability of the system generally, have been relieved by the flow, rendering the patient less liable to hysteritis and peritonitis. These facts, which may be deemed somewhat important, as connected with polypi and the time of removing them, I do not remember to have seen noticed by any writer.

NOTE.—Annexed, is a cut of the Chassaingnac ecraseur, as modified by J. W. Brooks, and used by him in the above case, and in others since. The improvement consisting in the curving of the staff, and a spring chain. *A*, the staff; *B*, the curve; *C*, the spring chain; *D*, the port-hole; *E*, the loop of chain, after passing out, supporting itself.

*Chicago, Dec. 8th, 1866.*

## Figures and Tables

**Figure f1:**